# Ethanol effect on metabolic activity of the ethalogenic fungus *Fusarium oxysporum*

**DOI:** 10.1186/s12896-015-0130-3

**Published:** 2015-03-12

**Authors:** Thomas Paschos, Charilaos Xiros, Paul Christakopoulos

**Affiliations:** Biotechnology Laboratory, School of Chemical Engineering, National Technical University of Athens, 9 Iroon Polytechniou Str, Zografou Campus, Athens, 5780 Greece; Industrial Biotechnology, Department of Chemical and Biological Engineering, Chalmers University of Technology, Kemivägen 10, Gothenburg, 41296 Sweden; Biochemical Process Engineering, Division of Chemical Engineering, Department of Civil, Environmental and Natural Resources Engineering, Luleå University of Technology, Luleå, SE–971 87 Sweden

**Keywords:** Bioethanol, Ethanol inhibition, Ethanol tolerance, Ethanol removal, *Fusarium oxysporum*

## Abstract

**Background:**

*Fusarium oxysporum* is a filamentous fungus which has attracted a lot of scientific interest not only due to its ability to produce a variety of lignocellulolytic enzymes, but also because it is able to ferment both hexoses and pentoses to ethanol. Although this fungus has been studied a lot as a cell factory, regarding applications for the production of bioethanol and other high added value products, no systematic study has been performed concerning its ethanol tolerance levels.

**Results:**

In aerobic conditions it was shown that both the biomass production and the specific growth rate were affected by the presence of ethanol. The maximum allowable ethanol concentration, above which cells could not grow, was predicted to be 72 g/L. Under limited aeration conditions the ethanol-producing capability of the cells was completely inhibited at 50 g/L ethanol. The lignocellulolytic enzymatic activities were affected to a lesser extent by the presence of ethanol, while the ethanol inhibitory effect appears to be more severe at elevated temperatures. Moreover, when the produced ethanol was partially removed from the broth, it led to an increase in fermenting ability of the fungus up to 22.5%. The addition of *F. oxysporum*’s system was shown to increase the fermentation of pretreated wheat straw by 11%, in co-fermentation with *Saccharomyces cerevisiae*.

**Conclusions:**

The assessment of ethanol tolerance levels of *F. oxysporum* on aerobic growth, on lignocellulolytic activities and on fermentative performance confirmed its biotechnological potential for the production of bioethanol. The cellulolytic and xylanolytic enzymes of this fungus could be exploited within the biorefinery concept as their ethanol resistance is similar to that of the commercial enzymes broadly used in large scale fermentations and therefore, may substantially contribute to a rational design of a bioconversion process involving *F. oxysporum*. The SSCF experiments on liquefied wheat straw rich in hemicellulose indicated that the contribution of the metabolic system of *F. oxysporum* in a co-fermentation with *S. cerevisiae* may play a secondary role.

## Background

*Fusarium oxysporum* is a filamentous fungus, capable of producing ethanol not only from hexoses but also from pentoses. The presence of many cellulases and hemicellulases in its secretome is reflected to the ability to grow on many lignocellulosic substrates under submerged or solid state conditions [1-3]. The fungus is capable of degrading and fermenting a wide variety of different substrates under anaerobic or limited oxygen conditions in the presence of inhibitory compounds such as furan derivatives, phenolic compounds and weak acids [4]. The low ethanol production rate and in some cases the formation of significant amount of acetic acid as by-product, have been considered as obstacles for its industrial exploitation. However, it has been shown that the efficient lignocellulolytic secretome of this fungus combined with its ability to ferment xylose could significantly improve the ethanol production from lignocellulosic substrates in co-culture with *Saccharomyces serevisiae* [5]. The beneficial properties of *F. oxysporum* can be fully exploited in consolidated bioprocess (CBP) where a microbial system able to degrade lignocellulose and ferment the released sugars in a single reactor is used. CBP is decreasing not only the overall cost but also the environmental impact of the process by minimizing the addition of commercial lignocellulolytic enzymes. The economics of the process can be further improved by operating at high gravity (HG) conditions (operating at total solids content above 20% w/v). Although HG technology is associated with challenges such as the insufficient mixing of the lignocellulosic slurries, the absence of significant amounts of free water and the presence of high amounts of microbial inhibitors, it has gained much attention as a promising technology due to the possibility to achieve final ethanol concentrations above 4% w/v [6]. The higher the ethanol concentration is, the lower the distillation cost (per g of ethanol produced) is [7]. The tolerance to ethanol of microorganisms used in such processes is therefore crucial for their use in large scale.

Ethanol affects the cellular membranes and influences cell metabolism and macromolecular biosynthesis by inducing the production of heat shock-like proteins, lowering the rate of RNA and protein accumulation [8], enhancing the frequency of petite mutations, altering metabolism, denaturing intracellular proteins and glycolytic enzymes and reducing their activity [9]. These inhibitory effects are reflected to the decreased cell division rate, to the decreased cell volume and to low specific growth rates, while high ethanol concentration reduces cell vitality and increases cell death [10]. Robust fermenting microorganisms like *S cerevisiae*, have developed appropriate mechanisms to deal with several types of damages caused by increased ethanol concentration. The yeast stress response is a transient reprogramming of cellular activities to ensure survival in challenging conditions, protect essential cell components and enable cells to resume their normal metabolic conditions [11].

Although most of the published articles on the field deal with yeasts’ ethanol stress and tolerance, there is some evidence concerning ethanol stress and cell response in fungi as well. The addition of ethanol to submerged cultures of *Phanerochaete chrysosporium* affected both the mycelial morphology and the fungal wall permeability and led to decreased pellet diameter and fungal biomass net weight [12]. The presence of ethanol (0.5–2%, v/v) hampered the secretion of cellulases by *Trichoderma reesei* [13]. The authors suggested that ethanol inhibition occurred at a pre-translational level by interfering with either the formation or the stability of cellulase mRNA. Asiimwe et al. reported indications that cytosolic aldehyde dehydrogenases play an important role in ethanol tolerance of the mycorrhizal fungus *Tricholoma vaccinum* [14]. The abundance of certain intracellular metabolites has also been connected to the fungal ethanol stress response: Evidence about the role of sterol glycosides and cerebrosides in the cell response to elevated ethanol levels was provided by the strong increase in the content of sterol glucoside that was observed after the treatment of fungal cells with increased ethanol concentrations [15].

Studying the effects of ethanol not only on the final yields but also on the biomass and ethanol rates is essential in order to evaluate a microorganism with regard to its implementation in biofuels production processes. Furthermore, the possibility of using lignocellulolytic activities in simultaneous saccharification and fermentation (SSF) processes is highly dependent on their stability during such a process. Ethanol is considered to be a non-competitive inhibitor for cellulases and this inhibition probably results from reversible enzyme denaturation [16]. Ethanol, like other organic molecules such as butanol or acetone, binds on the non-catalytic region of the enzyme, causing changes in the shape of the protein molecule, which in turn affects the catalytic activity. However, not all the enzymes are affected by ethanol in the same way. As shown by Chen and Jin the presence of ethanol had a positive effect on *β-*glucosidase from *Penicillium decumbens*, especially at temperatures above 40°C [17]. Therefore, the study of the effects of ethanol on the cellulolytic activities is of major importance for the evaluation of a cellulolytic system to be used in SSF processes for ethanol production.

Although many scientific reports have been published on both the hydrolytic and the fermentative performance of *F. oxysporum* on lignocellulosic substrates, little study has been published on the ethanol tolerance of this fungus. Early works on the fermentative performance of *F. oxysporum* refer to the effects of ethanol on this fungus claiming a relatively high ethanol tolerance but those statements were only based on the increase in ethanol concentration during the fermentation stage [18-20]. Recently, Hennessy et al., [3] showed that *Agrobacterium tumefaciens*-mediated transformation could be exploited as a tool to generate significant degrees of phenotypic diversity in *F. oxysporum* strain 11C in response to alcohol stress. In the same study, the importance of further investigation of the ethanol tolerance of this fungus is underlined.

The present work is studying for the first time the effects of ethanol on the cellulolytic and xylanolytic activities of *F. oxysporum*, on growth on glucose and xylose under aerobic conditions, and on ethanol production from glucose and xylose during micro-aerobic fermentation. This work complimented with the resent work of Hennessy et al., [3] gives to the scientific community a new prospect for the investigation of the lignocellulose degrading and C5, C6 fermenting fungus *F. oxysporum* as a potential CBP microorganism. Furthermore to overcome the ethanol limits, this study explored an alternative operating condition for fermentation with stepwise partial removal of ethanol. Finally, SSF under high dry matter conditions has been performed in order to explore the contribution of the metabolic system of *F. oxysporum* along with *S. cerevisiae* in ethanol production.

## Results

### Effect of ethanol on growth (aerobic conditions) and fermentative performance (micro-aerobic conditions) of *F. oxysporum*

#### Growth stage

**Figure 1:**
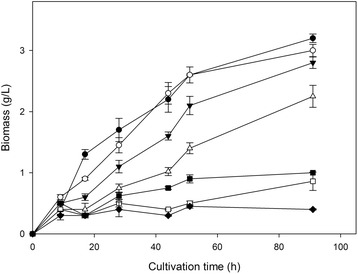
**Ethanol effect on** ***F. oxysporum*** **growth on glucose.** Ethanol was initially added at concentrations up to 6% w/v in the cultivations prior to inoculation, using glucose as carbon source. All experiments were carried out in duplicate, vertical bars indicate the error levels. The symbols stand for: No ethanol added (●), 1% w/v initial ethanol (○), 2% w/v initial ethanol (▼), 3% w/v initial ethanol (Δ), 4% w/v initial ethanol (■),5% w/v initial ethanol (□), 6% w/v initial ethanol (♦).

**Figure 2:**
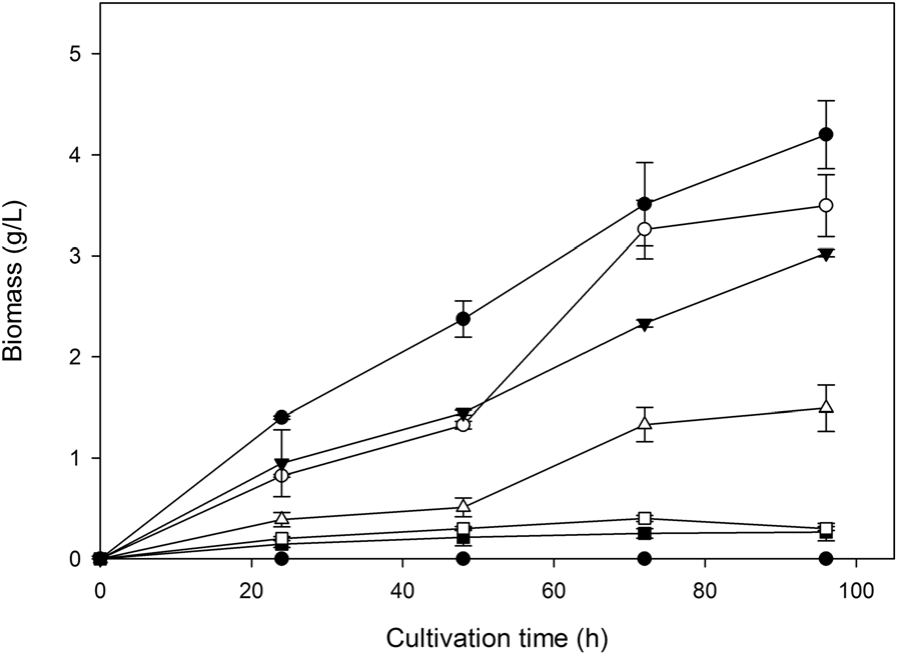
**Ethanol effect on** ***F. oxysporum*** **growth on xylose.** Ethanol was initially added at concentrations up to 6% w/v in the cultivations prior to inoculation, using xylose as carbon source. All experiments were carried out in duplicate, vertical bars indicate the error levels. The symbols stand for: No ethanol added (●), 1% w/v initial ethanol (○), 2% w/v initial ethanol (▼), 3% w/v initial ethanol (Δ), 4% w/v initial ethanol (■),5% w/v initial ethanol (□), 6% w/v initial ethanol (♦).

The ability of *F. oxysporum* to grow in the presence of ethanol under aerobic conditions was investigated. Various amounts of ethanol (0–60 g/L) were added to the medium prior to inoculation and the fungal growth was monitored over time. The effect of ethanol on growth on glucose was evident even 15 h after inoculation, while it was maximized after 44 h. As shown in Figure 1, the maximum biomass concentration decreased as the initial ethanol concentration increased. In the presence of 30 g/L of ethanol, the biomass produced corresponded to the 77% of the one in the absence of ethanol, while the decrease was dramatic when 40 g/L of ethanol were added to the medium. Almost no growth was observed when the experiments were performed in the presence of 60 g/L ethanol. The inhibitory effect of ethanol appears more severe when *F. oxysporum* was grown on xylose as carbon source. Figure 2 indicates that, the produced biomass was severely diminished when ethanol concentration exceeded 30 g/L and actually no growth was observed over 40 g/L initial ethanol in the broth.

Furthermore, the inhibitory effect of ethanol on fungal growth was clearly reflected not only on biomass yield but also on the growth rate. As shown in Table 1, the specific growth rate decreased dramatically as the ethanol concentration in the broth increased. In order to fully describe the kinetic pattern of ethanol inhibition on cell growth, the kinetic model proposed by Luong [21] was applied:

1 $$ \frac{\mu_i}{\mu_o}=1-{\left(\frac{P}{P_m}\right)}^a $$

Where, μ_0_ and μ_i_ are the maximum specific growth rate and maximum specific growth rate in the presence of ethanol respectively. P_m_ is the critical ethanol concentration above which cells cannot grow. The relationship between specific growth rate and critical ethanol concentration could be described by the ethanol tolerance index “α” proposed by the same model (a linear relationship for α = 1, a hyperbolic relationship when α > 1 and a parabolic relationship when α <1) [21]. The average specific growth rates were calculated and plotted against ethanol concentrations to estimate P_m_ and α of our experimental data, extracting values of 7.2% and 1.05 for P_m_ and α, respectively when glucose was used as substrate, and Pm = 3.8% and α = 2.5 when the fungus was grown on xylose.

**Table 1:** **Growth rates and lag phases of** ***F.oxysporum’*** **s aerobic growth in the presence and absence of ethanol**

**Ethanol% (w/v)**	**0**	**1**	**2**	**3**	**4**	**5**	**6**
Growth on glucose							
μ(h^-1^)	0.045 ± 0,002	0.038 ± 0.001	0.035 ± 0.003	0.033 ± 0.003	0.002 ± 0.003	0.009 ± 0.002	0.009 ± 0.003
Lag phase (h)	9	9	17	28	44	N/A	N/A
Growth on Xylose							
μ(h^-1^)	0.29 ± 0.003	0.030 ± 0.003	0.026 ± 0.002	0.017 ± 0.002	0.005 ± 0.001	N/A	N/A
Lag phase (h)	9	9	18	48	N/A	N/A	N/A

#### Fermentation stage

**Figure 3:**
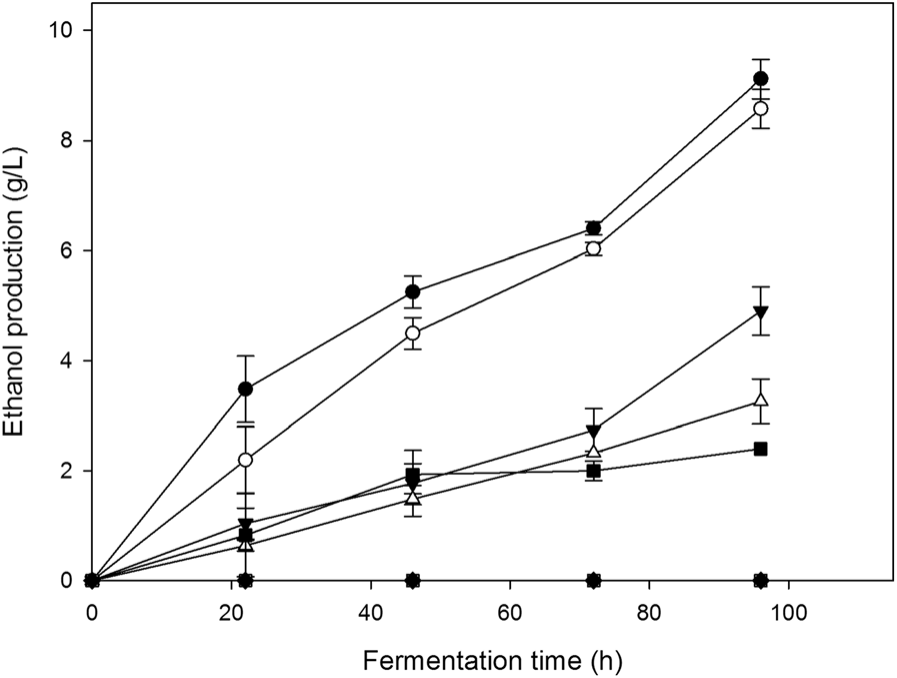
**Ethanol effect on** ***F. oxysporum*** **fermetative performance on glucose.** Ethanol was initially added at concentrations up to 6% w/v in the fermentation broth using glucose as carbon source. All experiments were carried out in duplicate, vertical bars indicate the error levels. The symbols stand for: No ethanol added (●), 1% w/v initial ethanol (○), 2% w/v initial ethanol (▼), 3% w/v initial ethanol (Δ), 4% w/v initial ethanol (■), 5% w/v initial ethanol (□), 6% w/v initial ethanol (♦).

**Figure 4:**
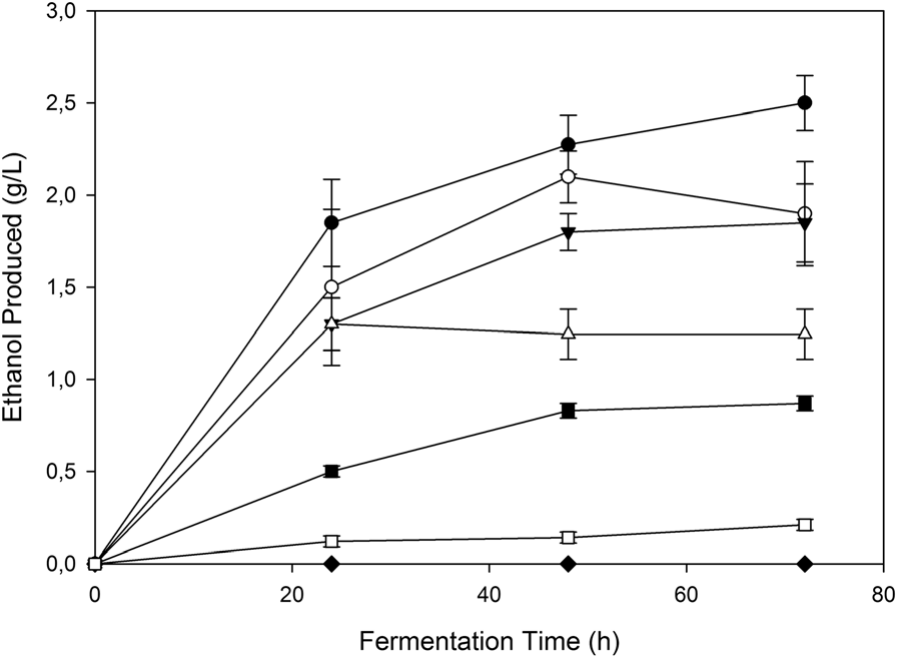
**Ethanol effect on** ***F. oxysporum*** **fermetative performance on xylose.** Ethanol was initially added at concentrations up to 6% w/v in the fermentation broth using glucose as carbon source. All experiments were carried out in duplicate, vertical bars indicate the error levels. The symbols stand for: No ethanol added (●), 1% w/v initial ethanol (○), 2% w/v initial ethanol (▼), 3% w/v initial ethanol (Δ), 4% w/v initial ethanol (■), 5% w/v initial ethanol (□), 6% w/v initial ethanol (♦).

In order to investigate the ethanol effect on the ability of the fungus to convert sugars to ethanol, *F. oxysporum* submerged cultivations previously grown under aerobic conditions, were subsequently shifted to micro-aerobic conditions, while various ethanol concentrations (0–6% w/v) were added at the start-up of fermentation stage. Figures 3 and 4 show the net ethanol amounts produced by *F. oxysporum* cells at different initial ethanol concentrations with glucose or xylose as carbon sources respectively. The total ethanol concentration to which the cells are exposed during the fermentation course was the initial amount added to the broth plus the produced ethanol. The experimental results obtained on glucose fermentation showed that both ethanol production rate and final ethanol concentration were affected by the initial ethanol level added in the culture. When glucose used as carbon source, the addition of 2%, 3% and 4% w/v ethanol in the culture media resulted in reduction of 46%, 65% and 74% in the net ethanol production, respectively, while no fermenting activity was observed after the addition of 5% or 6% (w/v) of ethanol. Using xylose as carbon source, the reductions in ethanol yields were 26%, 50% and 68% when 2%, 3% and 4% w/v ethanol was added with no observed ethanol production over 5% (w/v) of initially added ethanol.

### Effect of ethanol on the cellulolytic and xylanolytic systems of *F. oxysporum*

**Figure 5:**
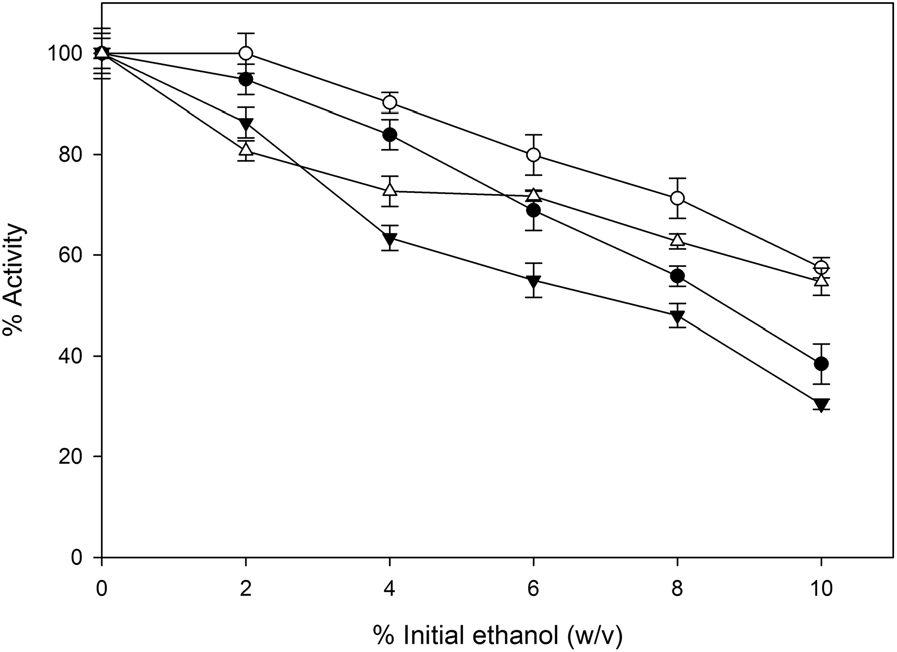
**Ethanol effect on cellulolytic enzymatic activities.** The activity assays were carried out under the addition of ethanol in the reaction buffer. *F. oxysporum* enzymes compared to mixture of commercial enzymes Celluclast 1,5 L – Novozyme 188. All assays were carried out in duplicate, vertical bars indicate the error levels. (▼) indicates *F. oxysporum* enzyme complex at 50°C and (Δ) at 30°C. (●) indicates commercial enzymes at 50°C and (○) at 30°C.

**Figure 6:**
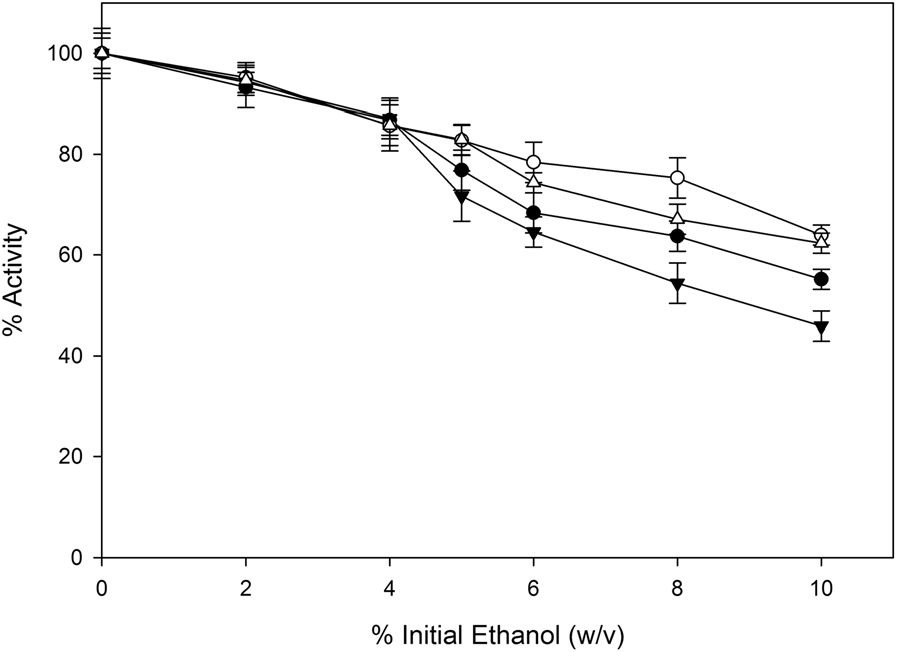
**Ethanol effect on hemicellulolytic enzymatic activities.** The activity assays were carried out under the addition of ethanol in the reaction buffer. *F. oxysporum* enzymes compared to mixture of commercial enzymes Celluclast 1,5 L – Novozyme 188. All assays were carried out in duplicate, vertical bars indicate the error levels. (▼) indicates *F. oxysporum* enzyme complex at 50°C and (Δ) at 30°C. (●) indicates commercial enzymes at 50°C and (○) at 30°C.

To evaluate the potential use of *F. oxysporum* cellulolytic system in consolidated or SSF bioprocesses, the effect of ethanol on the secreted hydrolytic activities was studied by measuring both the FPA and the xylanase activity in the presence of several ethanol levels. To evaluate the obtained data we performed similar studies using commercial enzymatic preparations. The results are shown in Figures 5 and 6. The assay reactions were carried out in two different temperatures, 30°C and 50°C. The first is the most likely temperature for a consolidated or SSF bioprocess, while the second is the standard temperature assay for the most lignocellulolytic enzymatic activities. The cellulolytic activity of *F. oxysporum* at 30°C was not significantly affected in ethanol concentrations up to 2% w/v (Figure 5). Moreover, despite the obvious reduction in higher ethanol concentrations, the residual activity at 6% and 8% (w/v) was measured at 72% and 63% of the initial, respectively. The ethanol inhibition was, as expected, more severe at 50°C where the enzymatic activity was reduced to 64% and 47% of the initial in the presence of 6% (w/v) and 8% (w/v) of ethanol, respectively. Finally, under the presence of 10% (w/v) ethanol the enzymes exhibited 50% and 70% of their initial activity at 50°C and 30°C, respectively. The decrease in cellulolytic activity showed a similar trend for both the commercial and the *F. oxysporum* enzyme mixtures (Figure 5).

The ethanol effect on xylanolytic activities is shown in Figure 6. Similarly with cellulases the hemicellulolytic activity of *F. oxysporum* was more intensely affected at 50°C. As shown, under most of the ethanol concentrations tested, the commercial mixture retained slightly higher activities (5% to 10%).

**Figure 7:**
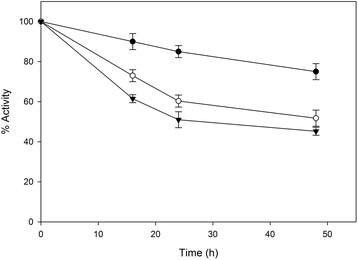
**Ethanol effect on lignocellulolytic enzymatic stabilities of** ***F. oxysporum*** **.** *F. oxysporum* enzymes were incubated at 30°C in the presence of 0% w/v (●), 2,5%w/v (○) and 5% w/v (▼) ethanol.

**Figure 8:**
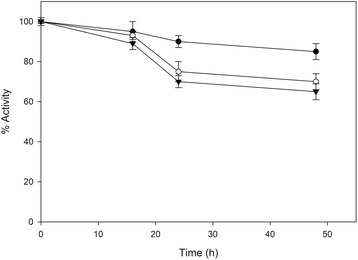
**Ethanol effect on lignocellulolytic enzymatic stabilities of commercial enzyme mixture.** The commercial enzyme system Celluclast 1,5 L – Novozyme 188 were incubated at 30°C in the presence of 0% w/v (●), 2,5%w/v (○) and 5% w/v (▼) ethanol.

The stability of the lignocellulolytic enzymes in the presence of ethanol is another important parameter regarding their suitability for SSF or CBP processes where they have to withstand elevated ethanol concentrations for many hours. Therefore, ethanol stability tests were performed using *F. oxysporum* enzymes in comparison with commercial enzymes at 30°C in the presence of 25 g/L and 50 g/L ethanol. As shown (Figures 7 and 8), commercial enzymes retained 70% and 65% of their activity at 2.5% and 5% of ethanol over 48 hours of incubation while *F. oxysporum* enzymes retained 52% and 45% of their activity under the same conditions. It is worth to be noticed that the commercially available enzymatic preparations often contain additives in order to increase their stability.

### Removal of ethanol during fermentation process

**Figure 9:**
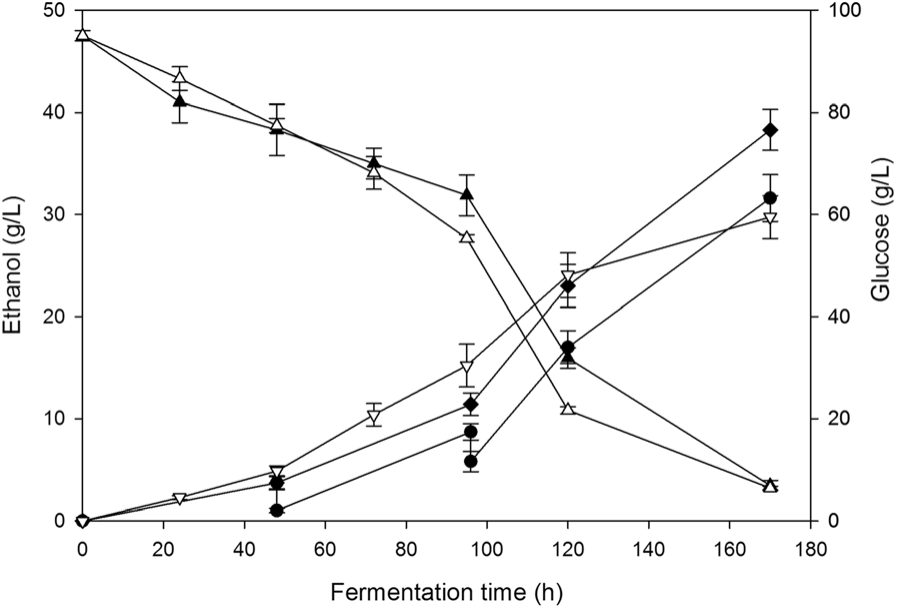
**Effect of ethanol removal during the fermentation process.** The produced ethanol during fermentation process was removed from the broth every 48 hours. All measurements were carried out in duplicate, vertical bars indicate the error levels. Ethanol present in broth (●), total ethanol produced (ethanol present in broth plus the removed ethanol) (♦), glucose consumption (▲), ethanol production in control experiment (∇) glucose consumption in control experiment (∆).

As proved in the present study (Figure 3 and 4), the presence of ethanol had an inhibitory effect on *F. oxysporum*’s fermenting ability. To examine whether the presence of ethanol has irreversible effects on the fungal metabolism, the ethanol was partially removed from the broth every 48 hours, as described in Methods section. Fermentation under vacuum has been performed in the past in order to overcome the inhibitory effects of ethanol [22,23]. Figure 9 shows that the ethanol production of *F. oxysporum* was increased by 22.5% when the ethanol was partially removed from the broth, showing that when the excessive ethanol was removed from the broth, *F. oxysporum* regained its metabolic activity confirming the reversible character of ethanol inhibition on *F. oxysporum* cells under the conditions of the experiment.

### Evaluation of *F. oxysporum* potential during co-fermentation of liquefied wheat straw

Finally, in order to test the hypothesis that *F. oxysporum* can be efficiently used in fermentation processes contributing in xylose fermentation and minimization of the amount of externally added enzymes by exploiting the hydrolytic enzymes produced by the fungus, 50 mg/g DM of *F. oxysporum* cells and/or 5 FPU/g DM enzymes were added along with *S. cerevisiae* in the start-up of fermentation stage. As control experiments, fermentations of the material with (a) *S. cerevisiae* and commercial enzymes, (b) *S. cerevisiae* and no addition of enzymes, (c) *S. cerevisiae* and *F. oxysporum* biomass, (d) *S. cerevisiae* and *F. oxysporum* enzymes were conducted. Results are presented in Table 2. It can be pointed out that the addition of *F. oxysporum* enzyme system can adequately replace the commercial enzyme system reaching 94% of the ethanol yield. Furthermore, when there was no enzyme addition at the startup of the fermentation process, the combined effect of both microorganisms resulted in a slight increase in ethanol production of about 7%, compared to the fermentation only with *S. cerevisiae.* Finally, the addition of *F. oxysporum*’s system in the SSF process increased the ethanol production by about 11%, compared to the fermentation conducted with *S. cerevisiae* and commercial enzymes.

**Table 2:** **Fermentation of liquefied pretreated wheat straw, with and without the addition of** ***F. oxysporum*** **’s system**

	**SSCF**	***F. oxysporum*** **enzymes**	***F. oxysporum*** **cells**	**Commercial enzymes**	**No additions in fermentation**
% DM	25	25	25	25	25
Enzymes at liquefaction	5 FPU/g DM	5 FPU/g DM	5 FPU/g DM	5 FPU/g DM	5 FPU/g DM
F. *oxysporum* enzymes added at fermentation	5 FPU/g DM	5 FPU/g DM	None	None	None
Commercial enzymes added at fermentation	None	None	None	5 FPU/g DM	None
F. *oxysporum* biomass	50 mg/g DM	None	50 mg/g DM	None	None
*S. cerevisiae*	5 mg/g DM	5 mg/g DM	5 mg/g DM	5 mg/g DM	5 mg/g DM
**Ethanol produced (g/L)**	**34.7 ± 1.1**	**29.2 ± 0.8**	**21.1 ± 1.0**	**31.2 ± 0.9**	**19.6 ± 0.8**

The addition of *F. oxysporum* biomass and enzyme system where tested for the fermentation of liquefied pretreated wheat straw. *F. oxysporum* enzymes were tested as a substitute of commercial enzymes and *F. oxysporum* biomass for its ability to cooperate with *S. cerevisiae* in ethanol production. SSCF stands for Simultaneous Saccharification and Co-Fermentation.

## Discussion

The results presented here showed that the response of *F. oxysporum* to ethanol can be compared to that of other xylose fermenting microorganisms used in bioethanol production processes. Studies on *Pichia stipites* showed that no growth was observed when the initial ethanol concentration was over 3.5% w/v during cultivation on glucose or xylose [24,25]. Additionally, there was no ethanol production measured on either source over the same ethanol concentration. Similarly, the growth of *Kloeckera africana* on Agave (tequila juice) at ethanol concentration above 2.5% w/v was associated with reduced biomass production [26]. Another study by Bajpai and Margaritis, made on *Kluyveromyces maxianus,* indicated that ethanol caused inhibitory effects on cell growth and fermentation ability [27]. As shown in that study, it was mainly the growth and ethanol production rates that were decreased, while the biomass and ethanol yields were almost not affected by the presence of ethanol. In accordance with the results presented here, a linear negative correlation of the specific growth rate with the initial ethanol concentration and a similar linear relationship between μ_max_ and ethanol concentration was reported [27].

The effects of ethanol on growth of *F. oxysporum* (aerobic conditions) were more severe when xylose was the carbon source in contrast with the ethanol effects in the productive anaerobic stage where the inhibition was stronger on glucose. The difference in the effects on the fermentative performance could be explained by the higher net ethanol production in the case of glucose. Although the net amount produced did not exceed 9 g/L, we must take into account the total ethanol levels. Furthermore, it has been proposed that the toxic effect of the endogenously produced ethanol to the microorganisms is more severe than the effect of the externally added ethanol [28,29]. However, other studies explained similar observations by the cumulative toxic action of ethanol together with other toxic by-products of fermentation, such as organic acids [30,31]. Although organic acids’ concentrations were not measured during the present study, it has been shown that acetic acid is a major by-product of the metabolism of *F. oxysporum* grown on xylose at similar conditions [32].

During the consolidated and SSF processes the need of an active enzyme system is necessary to maintain high saccharification rates and yields. *F. oxysporum* secrets a multi-enzyme system, rich in cellulases and hemicellulases, which is very efficient in biomass saccharification [33]. *F. oxysporum*’s enzymatic system showed an adequate tolerance to ethanol. Especially at 30°C, the most common temperature for SSF processes, (where *F. oxysporum*’s enzymatic system is more likely to be exploited) the effect of ethanol on the cellulolytic and xylanolytic activities of the enzymatic system was significantly less severe than at 50°C. Different reasons could explain this result. Various researchers have previously shown that ethanol reduces the enzymatic activities of cellulases. Its inhibition effect is temperature depended and increases as the reaction temperature increases [34,35]. Although the inhibitory effects of ethanol on cellulases are in many cases demonstrated studies have reported also destabilizing effects [17,36,37]. It seems that at elevated temperatures, the destabilizing effect of ethanol may accelerate denaturation of the enzyme even if the temperature itself could not cause protein denaturation for such a short period (as in our case, 1 h) [38]. Skovgaard and Jorgensen working on a set of thermo tolerant enzyme mixture found that the effect of ethanol on the activity reduction as the reaction temperature increases is not related to inhibition but rather to destabilization of the enzymes [39].

As shown, the ethanol effect on *F. oxysporum* enzymes is temperature dependent. When the assay is performed at 30°C, *F. oxysporum*’s cellulolytic and xylanolytic activity remains in high levels, in the presence of considerable amounts of ethanol. Even in the presence of 50 g/L of ethanol, *F. oxysporum’s* enzymes retain 45% of their activity over 48 hours. This could be beneficial in a consolidated bioprocess where the amount of commercial enzymes added in the hydrolysis step could be reduced by the ability of *F. oxysporum* to produce ethanol tolerant enzymes, able to continue the saccharification during the fermentation stage. Another study indicated that, *F. oxysporum* in mixed culture with *S. cerevisiae* was successfully used to ferment carbohydrates of wet exploded pre-treated wheat straw to ethanol under an SSF mode [5].

Additionally, when the produced ethanol was partially removed from the fermentation system of *F. oxysporum* in a stepwise manner the final ethanol production level was increased by around 23% reaching 38.4 g/L. The increase in ethanol production achieved here is similar to that reported in earlier studies dealing with the *in situ* removal of ethanol from the fermentation broth, where an increase up to 30% in the overall ethanol production could be achieved in most cases [22,40-42]. As shown in Figure 9, the beneficial effect of ethanol removal is clear only when the ethanol present in the medium is above 3% w/v. When ethanol concentration in the broth tends to reach 3%, the fermentation seems to stop. This is consistent with our findings regarding the ethanol tolerance levels of *F. oxysporum*. In cultures where the ethanol was partially removed it is obvious that this removal allowed the total ethanol production to exceed the 3% barrier. These results, suggest that as the ethanol concentration diminishes sufficiently, any metabolic inhibitory effects are reversed. As a result, the fermenting capacity is recovered when ethanol is removed. The ethanol concentration achieved here is very close to the crucial ethanol concentration of 4% (w/v) in the broth which is considered as a minimum prerequisite for a feasible large-scale distillation technology [7].

The straw used at the present work is rich in hemicellulose, and thus suitable to evaluate the ability of *F. oxysporum* not only to hydrolyze it but also to ferment the released xylose. The addition of *F. oxysporum* cells in the fermentation process resulted in 11% increase of the ethanol production (Table 2). Specifically, if ethanol yields are based on the dry material, the mixed microbial culture fermentation led to 138 g ethanol per kg pretreated wheat straw, while fermentation only with *S. cerevisiae* resulted in 128 g ethanol per kg pretreated wheat straw. The results shown in Table 2 indicate that *F. oxysporum*’s system is worthy of further investigation regarding its potential to enhance ethanol production during an SSF process along with *S. cerevisiae* when pentoses are present in the fermentation medium. These results are in accordance with Panagiotou et al. who reported in an earlier study that *F. oxysporum* multienzyme and microbial system had positive effect in ethanol production [5]. Moreno et al. [43] reported ethanol concentration 25 g/L at 20% solid content, which corresponds to 125 g ethanol per kg of pretreated material. Whereas, Jorgensen et al. [35] reported ethanol concentration up to 130 g/kg, applying a similar liquefaction process to that of the present study, in a high hemicellulose content material. At the present work, the liquefaction step took place for 6 hours, and 5 FPU/g DM were used at the liquefaction step supplemented with 5 FPU/g DM at the SSF process. Comparatively the enzyme loadings of Moreno et al., [43] and Jorgensen et al. [35] were 15 FPU/g DM and 7 FPU/g DM respectively. The saccharification times also vary, at the present study 6 hours of liquefaction were followed by 48 h of Simultaneous Saccharification and Co-Fermentation (SSCF), while Moreno et al. [43] conducted 144 h of SSCF and Jorgensen et al. [35] conducted 24 h of liquefaction and 48 h of SSF.

The complexity of SSCF process when dealing with lignocellulosic materials justifies the differences observed by different researchers. Many factors are affecting ethanol yields when operating at high solids fermentations, such as the nutrients deficiency, the presence of other inhibitors, the viscosity, and the inhomogeneity [6]. Moreover, differences between different raw materials and different pretreatments (The SF value of the pretreated wheat straw used in the present study was calculated at 3.58 (same as Jorgensen et al.), while Moreno et al. and presented a process of SF 3.34) make these processes difficult to control and to compare in many cases. Undoubtedly, many factors not being the objectives of this study, are affecting *F. oxysporum* ethanol yields and productivities and no clear conclusions regarding the contribution of *F. oxysporum* metabolic system to xylose fermentation during SSCF can be made.

Considering the above, the results of the present study show that *F. oxysporum*’s addition led to a significant ethanol increase, exploiting the hydrolytic enzymes produced by the fungus and thus reducing the amount of commercial enzymes needed. On the other hand, the ethanol yield, of about 31% of the maximum theoretical based on total carbohydrates content, achieved in the present work indicates that further investigation of the process is required.

## Conclusions

The results presented here assess three different aspects of *F. oxysporum* ethanol tolerance (effects on growth, enzymes, fermenting ability) for the use of a microorganism in a bioethanol production process and show that *F. oxysporum* can be used in a biorefinery both as a pentose fermenting and enzyme producing microorganism. Hemicellulose rich streams, produced after pretreatment of the raw material could be used for the production of necessary enzymes for the degradation of lignocellulosic biomass. Although the ethanol tolerance levels of *F.oxysporum* indicated that the fungus could be useful at the early stages of the fermentation, this was not confirmed by the SSCF and SSF results, in those cases *F. oxysporum* biomass did not demonstrate the expected contribution to the ethanol production, probably due to other stress factors not studied during this work (effect of high solids, presence of other inhibitory compounds). Nevertheless, the effect of *F. oxysporum* metabolic system could not be neglected as (even not as high as expected) the addition of *F. oxyporum* biomass increased the produced ethanol in an SSCF experiment along with *F. oxysporum* enzymes and *S. cerevisiae* compared to the one that only enzymes and yeast were added. The satisfactory ethanol tolerance levels of *F. oxysporum* enzymatic system allow the use of these enzymes also in high gravity bioconversion processes. This could lead to the reduction of the overall financial cost of the process, not only by achieving a high final ethanol concentration but also by reducing the amount of commercial enzymes added. To conclude with, this study as well as other studies made on *F. oxysporum* tolerance and adaptability [3,4] indicated that *F. oxysporum* is capable of dealing with ethanol, which makes it a very interesting microorganism for further study on CBP and SSCF processes.

## Methods

### Microorganism

*Fusarium oxysporum* F3 isolated from cumin [1], the fungus was grown on potato-dextrose-agar (PDA) slants for 5 days at 30°C. The slants were maintained as a stock culture at 4°C. Commercial dry baker’s yeast (Yiotis, Athens, Greece) was used at the pretreated wheat straw fermentations.

### Carbon sources – chemicals

Pretreated wheat straw (*Triticum aestivum* L) was used as raw material for ethanol production. The pretreatment of the straw (PWS) was performed in the Inbicon pilot plan at Skærbæk, Denmark. The residence time setpoint in the reactor was 12 min and the reactor temperature was maintained at 185°C by injection of steam. The severity effect (SF) was determined according to Garrote et al. [44] by the following equation:

$$ SF= \log (R)= log\left(t\cdot e\frac{T-100}{14.75}\right) $$

Pretreated wheat straw lignocellulose content was (%) cellulose 39.0 ± 1.5 and hemicellulose 21.0 ± 1.2. Structural carbohydrate content (cellulose and hemicellulose) was determined by a method adopted by the NREL protocol [45].

Brewers Grain (BG) and Corn Cobs (CC) were supplied by the Athens Brewery S.A. and the Agricultural University of Athens, respectively. BG composition was analyzed by Xiros et al. [46] and Corn Cobs was analyzed by Katapodis and Christakopoulos [47].

All chemicals and reagents were provided by Sigma-Aldrich (USA).

Commercial enzymes Celluclast 1.5 L and Novozyme 188 were provided by Novozymes (Denmark). The commercial enzymes were used in a 5:1 v/v ratio.

### Inoculum

For inoculum production, spores were extracted from the stock slants using 5 ml of sterile distilled water and then cultivated in 250 mL Erlenmeyer flasks containing 100 mL of the following mineral medium:(in g L^-1^) 1.00 KH_2_PO_4_, 0.30 CaCl_2•_2H_2_O, 0.30 MgSO_4•_7H_2_O, 10.00 (NH_4_)_2_HPO_4_, 6.94 NaH_2_PO_4° _2H_2_O, 9.52 Na_2_HPO_4° _2H_2_O [2], supplemented with either 40 g L^-1^ BG-CC (2/1) or 20 g L^-1^ glucose or xylose, depending on the experimental procedure. The pH was adjusted to 6.0. The flasks were incubated at 30°C for 2 days in an orbital shaker at 200 rpm (Zhicheng ZHWY-211C) for mycelium production.

### Aerobic submerged cultivation

Aerobic submerged cultures were carried out in 250 mL Erlenmeyer flasks containing 2 g of glucose or xylose and 100 mL of the above described mineral medium (pH 6.0). Medium and sugars were sterilized separately at 109°C for 40 min. The culture medium was inoculated with 10 ml of 48 h-old inoculum (prepared as described above). The flasks were incubated at 30°C for 7 days at 200 rpm. The flasks were capped with cotton that allowed aerobic conditions and assured sterile conditions. At different time intervals aliquots were aseptically withdrawn and used for biomass and sugar consumption estimation.

For analyzing the potential impact, of the presence of ethanol, on the growth of *F. oxysporum* the following experiment was carried out. In submerged cultures as described above ethanol concentrations up to 6% w/v were exogenously added in the broth. At different time intervals aliquots were aseptically withdrawn and used for biomass and sugar consumption estimation.

### Biomass estimation

Samples of the aerobic cultures were filtered using 0.2 μm pure size filter paper, Millipore (USA). The biomass content was measured by weighing the dried samples.

### Enzyme production

Crude enzyme extract production was carried out in 3 L Erlenmeyer flasks containing 40 g of carbon sources (BG-CC 2/1) and 1 L of mineral medium. Prior to sterilization, the initial pH of the medium was adjusted to 6.0. The medium was sterilized at 121°C for 20 min and was inoculated with 100 mL of 72 h old inoculum (prepared as described above). The flasks were incubated at 30°C for 5 days at 200 rpm. At the end of the enzyme production stage, the culture was centrifuged (14,000 rpm, 4°C, 40 min) and the clarified supernatant was concentrated using ultra filtration membranes (10,000 kDa) Millipore (USA), there was no buffer addition during ultrafiltration process.

### Enzyme assays

One unit (U) of enzyme activity was defined as the amount of enzyme required to liberate 1 μmol of product per minute, at assay temperature.

Filter paper activity (FPA) was determined as described by Wood and Bhat [48]. In a capped 2 ml Eppendorf tube, 1 mL of buffer solution, 0.5 mL of enzyme (with the appropriate dilution so to release less than 1 mg product per mL) and a filter paper (Watman #1 1x6 cm, approximately 50 mg) were added. The mixture was incubated at 50°C for 1 hour at 1000 rpm. The released reducing sugars were measured by the 3,5-dinitrosalicylic acid (DNS) method [49].

The β-1,4-d-endoxylanase activity was determined by incubating the enzyme for 10 minutes at 50°C with 1% birchwood xylan [50]. The released reducing sugars were measured by the 3,5-dinitrosalicylic acid (DNS) method [49].

### Ethanol effect on enzymatic activity

For the investigation of the effect of ethanol concentration on the cellulolytic and xylanolytic activity of the enzymes used in this study, assays were carried out in the presence of initial ethanol concentrations up to 10% w/v. The ethanol was added in the reaction buffer solution prior to the enzyme addition. The assay reactions were carried out as described above at either 30°C or 50°C.

### Ethanol stability

The ethanol stability of the enzymes was performed at 30°C the SSF temperature at which the enzymes withstand the presence of ethanol for many hours. The enzymes were incubated at 30°C with the addition of two ethanol concentrations, 25 g/L and 50 g/L. At different time intervals aliquots were taken and the cellulolytic activity was measured by the Filter Paper method.

### Analytical methods

Reducing sugars concentration was determined according to dinitro-3.5-salicilic acid (DNS) method [47]. The effect of ethanol on the sugar measuring method was determined by control measurements, containing all the ethanol concentrations used and a standard sugar concentration. Ethanol appeared to have no effect on the reducing sugar determining method. Glucose was measured according to commercial enzyme solution of GOD/PAP (glucose oxidase/ peroxidase assay) (Biosis, Greece). Xylose was measured according to commercial D-xylose assay kit (Megazyme, Ireland) Ethanol was analyzed using an HPLC system (Szimadju) equipped with an Aminex HPX-87H column (Bio-Rad, 300 x 7.8 mm, particle size 9 μm) using a Refractive Index (RI) detector. Mobile phase was 5 mM H_2_SO_4_ in HPLC grade water at 0.6 mL/min flow rate, column temperature was 40°C, injection volume was 50 μl and total runtime was 30 min. All samples were filtered (0,2 μm, Macherey-Nagel) prior to the analysis.

### Ethanol production

Ethanol production experiments under micro aerobic conditions were carried out in 250 mL Erlenmeyer flasks containing 2 g of glucose or xylose, sterilized at 109°C for 40 min. 100 mL of culture previously grown on glucose as carbon source for about 4 days (until substrate was fully consumed) at 30°C, were aseptically transferred in the Erlenmeyer flasks provided with needle-pierced rubber stoppers, which ensured micro-aerobic conditions and allowed the release of produced carbon dioxide [51] and the cultures were incubated at 30°C for 7 days and 80 rpm (Zhicheng ZHWY-211C). At different time intervals, aliquots were aseptically withdrawn and used for ethanol production and substrate consumption estimation.

For analyzing the potential impact, of the presence of ethanol, on the fermenting ability of *F. oxysporum,* micro aerobic cultivations of *F. oxysporum* were carried out, as mention before, in the presence of extracellular, exogenously added ethanol. The initial ethanol concentrations were up to 6% w/v. At different time intervals aliquots were aseptically withdrawn and used for ethanol and substrate consumption estimation.

All ethanol production experiments were carried out as described by Xiros et al. [4], Dogaris et al. [51] and Matsakas and Christakopoulos [52]. After each sampling, nitrogen (0.1 vvm) was flushed in the cultures for 10 minutes to assure the anaerobic conditions.

### Partial ethanol removal

Ethanol production experiments under micro aerobic conditions were carried out in 250 mL Erlenmeyer flasks containing 9.5 g of glucose sterilized at 109°C for 40 min. 100 mL of culture previously grown with glucose as carbon source for about 4 days (until glucose was fully consumed) at 30°C, were aseptically transferred in the Erlenmeyer flasks and the cultures were incubated at 30°C for 7 days at 80 rpm (Zhicheng ZHWY-211C). Every two days the broth was aseptically centrifuged and transferred to a rotary evaporator (Rotavapor RE 11, Buchi (Switzerland)) where the contained ethanol was partially removed. The vacuum process was carried out at 75°C for approximately 10 minutes. Consequently, the broth was returned in the Erlenmeyer flasks along with the centrifuged fungal cells. At this stage, nitrogen was flushed in the cultures to assure the anaerobic conditions. For avoiding broth condensation, the loss of water during the evaporation process was estimated by weighting. Equal amount of sterile water (less than 3 mL each time) was added in the culture.

### Liquefaction of hydrothermaly treated wheat straw

For the liquefaction and saccharification of the pretreated wheat straw a reactor was manufactured in house. The reactor consists of a cylinder drum 25 cm wide and 60 cm diameter, and a rotating shaft for mixing the material. A 0,55 kW motor was used for the rotation of the shaft. The rotation speed could be controlled from 0 up to 20 rpm, and the motor can be programmed to shift its rotation clockwise and anti-clockwise. An external jacket filled with oil was used for temperature control up to 90°C. The experiments were executed at 50°C, using 30% DM of straw supplemented with buffer (phosphate-citrate, 50 mM, pH 5,5) and an enzyme mixture of 5 FPU per g DM, consisting of Celluclast 1.5 L and Novozyme 188 (both from Novozymes A/S, Bagsværd, Denmark) in ratio of 5:1 v/v. The activity of this mixture was measured at 81 FPU/ml by the filter paper assay. The mixing speed was 7 rpm shifting clockwise and anti-clockwise twice a minute. The duration of the liquefaction was 6 h.

### Fermentation of liquefied wheat straw

The liquefied at 30% DM material was used as carbon source for ethanol production. *S. cerevisiae* (Dry Baker’s Yeast) and *F. oxysporum* (previously grown on glucose for 4 days under submerged cultivation) were used as the fermentative microorganisms. 5 mg/g DM dry yeast and 50 mg/g DM *F. oxysporum* centrifuged cells were added. Moreover 5 FPU/g DM of either commercial enzymes (Celluclast 1.5 L and Novozym 188) or *F. oxysporum’s* enzymes were added to enhance the hydrolysis. The final DM in the mixed fermentation was 25% w/w due to the addition of the enzymes and the fungal biomass. All experiments were carried out, in duplicate, under submerged cultivation at pH 5.5 and T = 30°C in 250 mL Erlenmeyer flasks.
